# Washed microbiota transplantation improves symptoms and intestinal barrier function in patients with functional bowel disorders: a propensity-score matching analysis

**DOI:** 10.1186/s12876-024-03131-z

**Published:** 2024-01-23

**Authors:** Xiao-Yan Ye, Jun-Yi Chen, Li-Hao Wu, Dan-Ping Luo, Xiao-Huo Ye, Li-Quan Wu, Xing-Xiang He

**Affiliations:** 1 Department of Gastroenterology, The First Affiliated Hospital of Guangdong Pharmaceutical University, 19 Nonglinxia Road, Yuexiu District, 510030 Guangzhou, Guangdong Province China; 2 Department of Gastroenterology, Research Center for Engineering Techniques of Microbiota -Targeted Therapies of Guangdong Province, 510030 Guangzhou, Guangdong Province China; 3 Department of Pharmacy, Heyuan Health School, 517000 Heyuan, Guangdong Province China

**Keywords:** Functional bowel disorders, Washed microbiota transplantation, Intestinal barrier function, Propensity-score matching, Cox regression analysis

## Abstract

**Background:**

Alterations in the intestinal microbiota may play a role in the pathogenesis of functional bowel disorders (FBDs). Probiotics are widely used to improve intestinal dysbacteriosis in FBDs. In the context of FBDs, washed microbiota transplantation (WMT) appear to be a promising therapeutic option. We aimed to compare probiotics with WMT by using a propensity-score matching analysis (PSMA).

**Methods:**

We conducted a retrospective investigation of 103 patients with FBDs, including irritable bowel syndrome (IBS), functional constipation (FC), functional diarrhea (FDr), functional abdominal bloating (FAB). Patients were divided into the WMT group or probiotics group (taking probiotics capsules). Data on the following parameters were matched for PSMA: age; sex; disease course; body mass index; anxiety; insomnia; tobacco smoking; alcohol consumption; and levels of D-lactate, diamine oxidase, and lipopolysaccharide. Intestinal barrier function (IBF) and symptoms were evaluated both before and after treatment initiation. Prognostic factors were assessed by Cox proportional hazards regression analysis.

**Results:**

PSMA identified in 34 matched pairs (11 IBS, 12 FC, 7 FDr, and 4 FAB in the probiotics group and 14 IBS, 13 FC, 5 FDr, and 2 FAB in the WMT group. Improvement of FBD symptoms was greater with WMT than probiotics (*P* = 0.002). The WMT group had significantly fewer patients with intestinal barrier damage than the probiotics group (38.2% vs. 67.6%, *P* = 0.041). This improvement of FBD with WMT was further reflected as a reduction in D-lactate levels (*P* = 0.031). Increased D-lactate levels which were identified as a prognostic factor for FBDs (HR = 0.248, 95%CI 0.093–0.666, *P* = 0.006) in multivariate Cox regression analysis.

**Conclusion:**

WMT could improve symptoms and IBF in patients with FBDs. Increased D-lactate levels in patients with FBDs may predict a favorable response to WMT treatment.

## Background

The Rome IV criteria define five functional bowel disorders (FBDs) according to the presenting symptoms: irritable bowel syndrome (IBS), functional constipation (FC), functional diarrhea (FDr), functional abdominal bloating/distension (FAB/FAD), and unspecified FBD. The pathogenesis of FBDs has been mainly attributed to gut–brain interactions, including altered gastrointestinal motility [1], visceral hypersensitivity [2], increased intestinal permeability [3], and altered intestinal flora [4]. However, recent studies have suggested that alterations in the intestinal microbiota may also play a role in the pathogenesis of FBD [5, 6], in that they may at least have the potential to affect intestinal functions in a manner relevant to the development of functional intestinal symptoms. Currently, probiotics are used to improve intestinal dysbacteriosis in patients with FBDs. Several randomized control trials have recently compared the effects of probiotics and placebos in FBDs. However, most of these studies have focused on IBS [7–11], and only a few have investigated FC [12–14], FDr [15, 16], and FAB [15, 17, 18]. The conclusions of the latter studies have also been inconsistent: some studies have shown probiotics to be superior to placebo, whereas others have shown comparable effects of the two.

Fecal microbiota transplantation (FMT) is a method used to rebuild the gut microbiome. FMT is recently being explored as a means to restore intestinal homeostasis in FBDs, particularly IBS and FC. However, data on the use of FMT in the treatment of FDr and FAB are scarce. Since its introduction in 2014, an automatic washing process has been used to prepare fecal microbiota from stool for use in FMT centers in China. FMT performed using this process is called “washed microbiota transplantation” (WMT) [19]. Randomized controlled trials have shown that certain probiotic products have a beneficial effect on relevant physiological factors in patients with IBS, such as the function of the mucosal barrier [20].

No studies have been hitherto examined whether WMT influences intestinal barrier function (IBF) in individuals with FBDs. This study was aimed at assessing the therapeutic effects of WMT in patients with FBDs in comparison with the traditional treatment of probiotics.

## Methods

### Study design and patients

In this retrospective study, we enrolled patients aged 18–80 years diagnosed with FBDs according to Rome IV criteria who were referred to the gastroenterology inpatient clinic of the First Affiliated Hospital within Guangdong Pharmaceutical University (Guangzhou, China) between 2017 and 2019.

Patients were assigned to two groups depending on the treatment they received: one group received WMT (WMT group) over two monthly courses, while the other received probiotics. All patients who received WMT treatment provided written informed consent. Patients in probiotics group were treated with probiotics capsules (a mixed preparation containing *Lactobacillus acidophilus* and *Bifidobacterium*).

### Inclusion and exclusion criteria

The criteria for inclusion in this study were as follows: (i) diagnosis of IBS, FC, FDr, or FAB on the basis of Rome IV criteria; (ii) gastroscopy, colonoscopy, and IBF assessment performed during the pretreatment evaluation; and (iii) IBF assessment performed during the post-treatment follow-up visit. Patients were excluded if they (i) had severe heart or lung disease; (ii) had liver or kidney failure; (iii) had a malignant tumor; (iv) were pregnant; (v) had undergone abdominal surgery; (vi) had other diseases that significantly affected quality of life; or (vi) refused/failed to complete follow-up.

### WMT Procedure

The WMT procedure was performed in accordance with the Nanjing Consensus on Methodology of Washed Microbiota Transplantation [21]. Prior to collection of stool samples, all healthy stool-donors aged 18–25 years were screened thoroughly via interviews, psychological and physical examinations, biochemical testing, and screening tests for infectious diseases. Stools required for WMT were extracted from the donated excrement. Samples were centrifuged at 2500 rmp for 3 min at room temperature and suspended thrice using an intelligent microbial-separation system (GenFMTer; FMT Medical, Nanjing, China). Five steps of filtration were carried out according to the manufacturer’s instructions. The transplants were transferred to patients within 1 h of preparation through a nasal jejunal feeding tube or transendoscopic enteral tubing. Patients who received WMT underwent two courses of transplantation (one course per month). In each course, 200 mL of bacterial solution was administered once daily for 3 days.

### Data collection

Demographic and laboratory data were collected from the medical records of the patients or the hospital database. Follow-up data were collected from electronic medical records and/or standardized telephonic interviews. All patients had undergone evaluation at the initial pre-treatment visit as well as during follow-up after treatment.

Effectiveness of WMT or probiotics in the treatment of IBS, FC, FDr, and FAB was assessed using the Irritable Bowel Syndrome-Severity Scoring System (IBS-SSS), Wexner Scoring System (WSS), frequency of stools per day, and Gastrointestinal Symptom Rating Scale (GSRS), respectively. Stool was assessed using the Bristol Stool Form Scale (BSFS). In addition, the occurrence of adverse effects of treatment were also documented. To assess IBF, we obtained measurements of the serum levels of diamine oxidase (DAO), D-lactate, and lipopolysaccharide (LPS), both before and after treatment initiation.

### Outcome measurement

Outcome measurements were evaluated in terms of clinical efficacy classified as “ineffectiveness,” “remission,” and “cure” as well as measures of biochemical markers of IBF. For IBS, remission was defined as symptom relief, as indicated by a score of > 75 points in the IBS-SSS or improvement of grade ≥ 1 in the Bristol School Form Scale (BSFS), while cure was defined by symptom relief, as indicated by a score of > 100 points in the IBS­SSS and grade 4 in the BSFS. For FC, remission was defined as a decrease in the WSS score by 30–69% or an increase of ≥ 1 grade in the BSFS, whereas cure was defined as a decrease in the WSS score by > 70% and grade 4 in the BSFS. For FDr, remission was defined by a decrease in the BSFS by > 1 grade or a reduced frequency of stools per day, while cure was defined by grade 4 in the BSFS and 1–2 stools per day. For FAB, remission was defined by a decrease in the GSRS score by 50–80%, whereas cure was defined as a decrease in the GSRS score by > 80%.

IBF was assessed on the basis of measurements of biochemical markers coupling of IBF, which include serum levels of DAO, D-lactate, and LPS determined using the tests developed by the Institute of Biophysics within the Chinese Academy of Sciences (Beijing, China) and manufacturer protocols. DAO, D-lactate, and LPS serum levels of > 10 U/L, > 15 mg/L, and > 20 U/L, respectively, indicated damage to the intestinal mucosa and increased intestinal permeability.

### Statistical analysis

Statistical analyses were performed using SPSS 26.0 (IBM, Armonk, NY, USA) and Prism 8 (GraphPad, San Diego, CA, USA). The results were expressed as frequencies and percentages for categorical variables. For continuous variables, the mean and standard deviation were given in case of a normal distribution, and medians and interquartile ranges were given in case of non-normal distribution. The Fisher’s exact test was used to analyze categorical variables. The Wilcoxon matched-pair signed-rank test was employed when analyzing paired data. A *P* value of < 0.05 (two-tailed) was considered statistically significant. Patients undergoing WMT were matched 1-to-1 with patients taking probiotics based on the propensity score using the nearest neighbor-matching method.

In addition, multivariate Cox regression analyses were performed to evaluate the associations between clinical outcomes and certain clinical characteristics, namely, age; sex; course; body mass index; anxiety; depression; insomnia; tobacco smoking; alcohol consumption; and serum levels of DAO, D-lactate, and LPS, in the propensity score-matched cohort.

## Results

### Propensity-score matching

Before propensity-score matching, the probiotics group contained older patients and more individuals with a history of alcohol consumption than those in the WMT group. The probiotics group comprised 42 patients, whereas the WMT group comprised 61 patients. After propensity-score matching, 34 matched pairs (34 cases from each group) were generated. A power analysis demanded that 34 patients are required in each group to produce a power of 90% and a *P* value of 0.05. There were no significant differences in the clinical and laboratory characteristics at baseline for the matched pairs of patients. In the probiotics group, 11 patients had IBS, 12 had FC, seven had FDr, and four had FAB and the mean ± SD interval between the diagnosis of FBDs and IBF assessment was 14.41 ± 8.79 months. In the WMT group, 14 patients had IBS, 13 had FC, five had FDr, and two had FAB, and the interval between FBDs diagnosis and IBF assessment was 2.66 ± 4.34 months (Table 1).

### Clinical outcomes

**Fig. 1:**
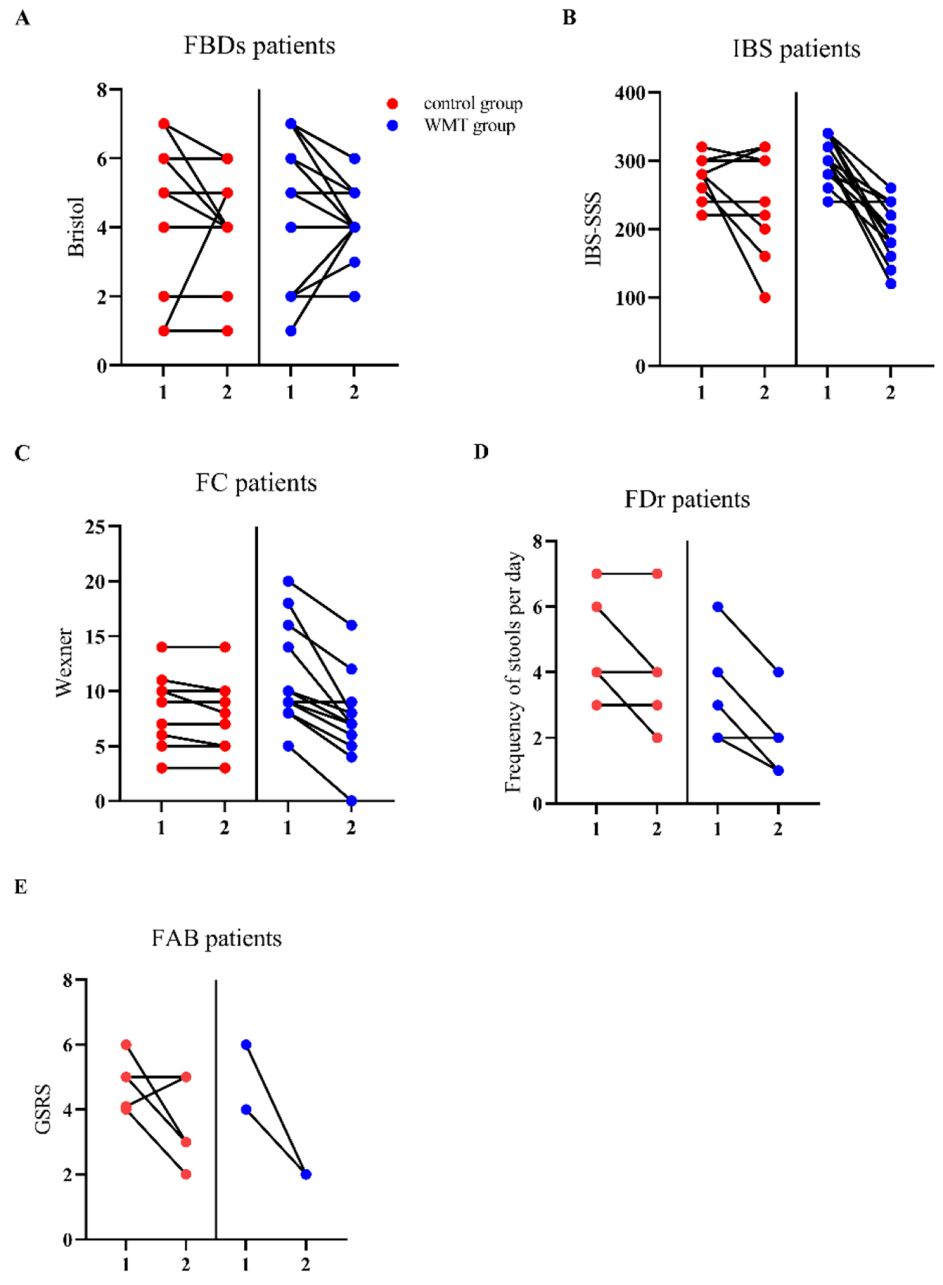
Changes in various scoring systems of FBD patients from the pre-treatment visit to follow-up visit (**A**) Changes in the Bristol Stool Form Scale grade of FBD patients in probiotics and WMT groups;(**B**) Changes in IBS-SSS of IBS patients in both groups; (**C**) Changes in the Wexner Scoring System score of FC patients in both groups; (**D**) Changes in the frequency of stools per day of FDr patients in both groups; (**E**) Changes in the GSRS score of FAB patients in both groups

**Fig. 2:**
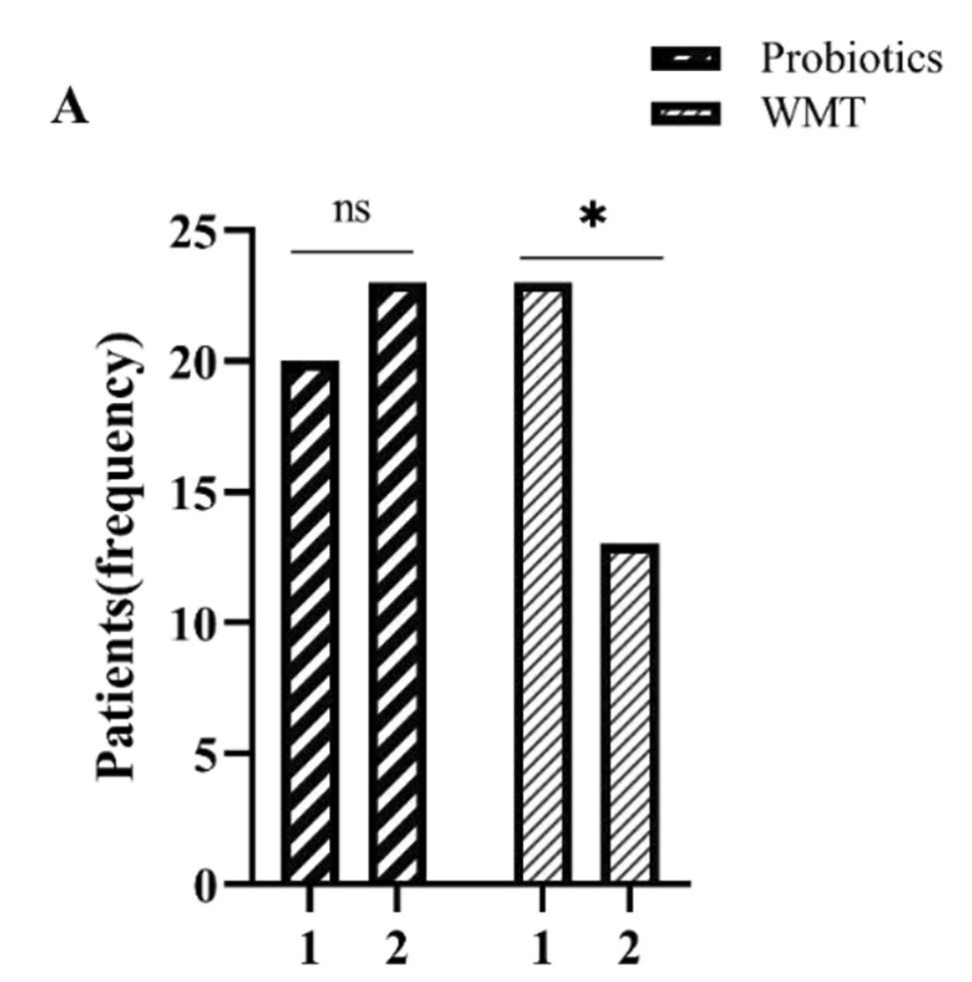
Changes in function of the intestinal mucosal barrier in FBD patients from the pre-treatment visit to follow-up visit in probiotics and WMT groups. ^∗^*P* < 0.05, ^ns^*P* > 0.05 (not significant)

**Fig. 3:**
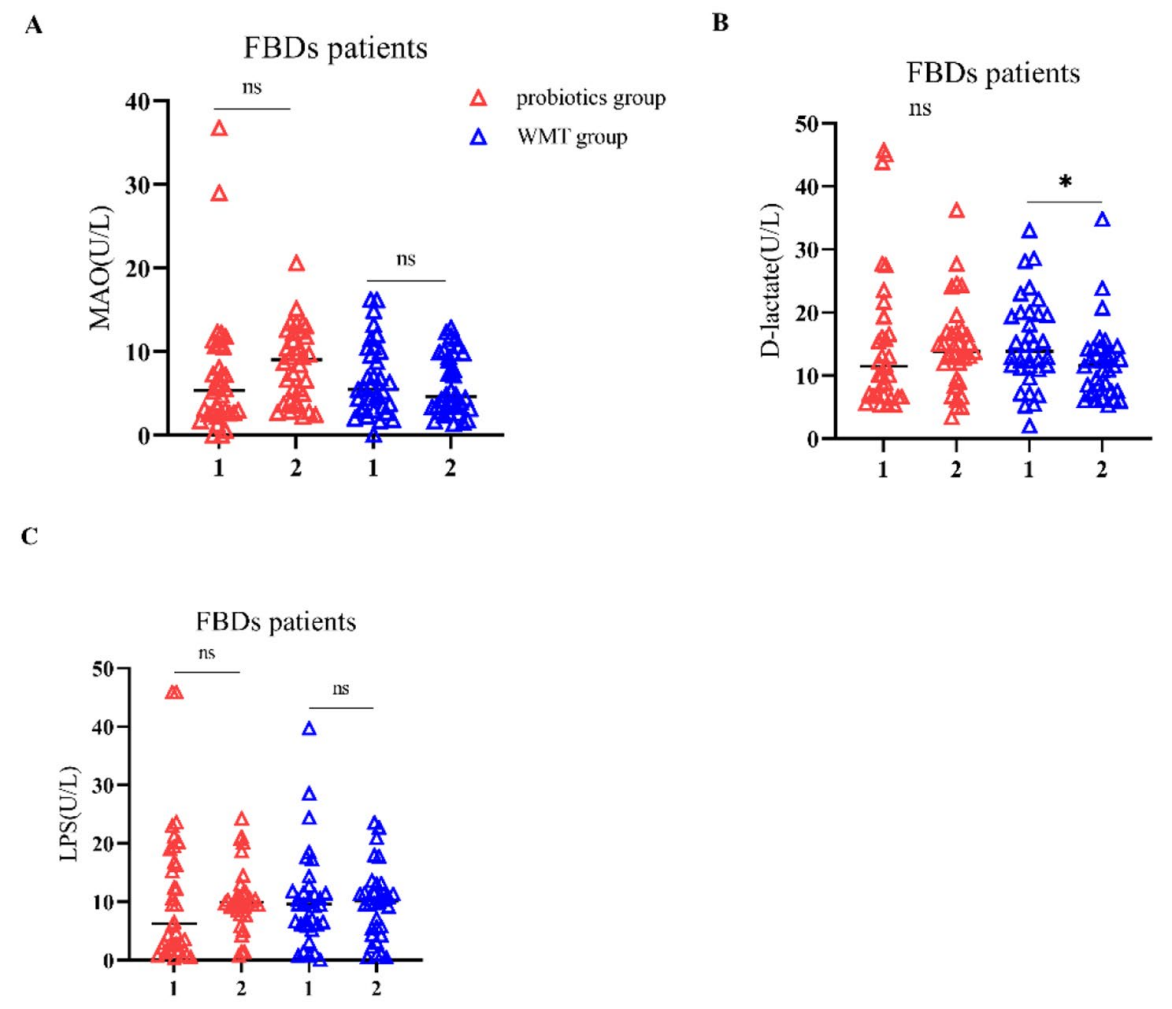
Changes in function of the intestinal mucosal barrier from the pre-treatment visit to follow-up visit. (**A**) Changes in MAO levels in probiotics and WMT groups; (**B**) Changes in D-lactate levels in both groups; (**C**) Changes in LPS levels in both groups. ^∗^*P* < 0.05, ^ns^*P* > 0.05 (not significant)

After treatment, cure was achieved in 26.5% (*n* = 9) and 14.7% (*n* = 5), whereas remission occurred in 55.9% (*n* = 19) and 26.5% (*n* = 9) of the patients in the WMT and probiotics groups, respectively. Compared with the probiotics group, the WMT group showed better results in all scores for clinical symptoms (Fig. 1), indicating that WMT was more effective than probiotics (χ^2^ = 12.253, *P* = 0.002) in relieving symptoms (Table 2). The probiotics group showed no obvious difference in the number of patients with evidence of damage to the intestinal barrier at the pretreatment and follow-up visit (58.8% vs. 67.6%, *P* = 0.648). In contrast, the WMT group showed a significant reduction in this regard (67.6% [i.e., 23 patients] vs. 38.2% [i.e., 13 patients]) (χ^2^ = 4.050, *P* = 0.041) (Fig. 2). Furthermore, in the probiotics group, there were no significant differences between the levels of DAO, D-lactate, and LPS at the pre-treatment and follow-up visit (*P* > 0.05). On the other hand, in the WMT group, the median level of D-lactate of 13.8 U/L (interquartile range, 11.12–19.80 U/L) at the pretreatment visit was markedly reduced to 11.55 U/L (interquartile range, 7.51–14.24 U/L) at the follow-up visit (Z = − 2.154, *P* = 0.031). However, this decrease was not observed in the case of the median levels of LPS and DAO, which remained similar both before and after treatment (*P* > 0.05) (Table 3; Fig. 3).

### Multivariate cox regression analysis

Twelve factors, namely, age; sex; disease course; body mass index; anxiety; depression; insomnia; tobacco smoking; alcohol consumption; and levels of DAO, D-lactate, and LPS, were evaluated for their prognostic value in FBDs. These factors were included in the multivariate Cox proportional hazards model for analyses. The hazard ratios (HRs) of the estimated poor prognosis risk based on WMT use are shown in Table 4. Increased levels of D-lactate before treatment initiation were found to be significantly associated with a lower risk of a poor prognosis (HR, 0.248; 95% confidence interval, 0.093–0.666, *P* = 0.006). HRs for other factors were not significant.

**Table 1:** Baseline clinical and laboratory characteristics

	Before propensity-score matching			After propensity score matching		
	Probiotics	WMT	*P*	Probiotics	WMT	*P*
	(*n* = 42)	(*n* = 61)		(*n* = 34)	(*n* = 34)	
Age (years)	63.00 (57.5–69.5)	58.00 (41.00–64.50)	0.001	63 (55.75–69.5)	62 (57.75–68.25)	0.740
Sex						
Male	24 (57.1%)	29 (47.5%)	0.340	16 (47.1%)	14 (41.2%)	0.625
Female	18 (42.9%)	32 (52.5%)		18 (52.9%)	20 (58.8%)	
Disease course (years)	2.00 (1.00–8.00)	5.00 (1.00–9.00)	0.172	3.00 (1.00–8.50)	5.00 (0.90–10.00)	0.893
BMI (kg/m^2^)	23.20 ± 3.01	22.29 ± 4.21	0.234	22.90 ± 2.81	22.62 ± 2.97	0.697
Anxiety	4 (9.5%)	13 (21.3%)	0.113	3 (8.8%)	2 (5.9%)	1.000
Depression	2 (4.8%)	6 (9.8%)	0.344	1 (2.9%)	0 (0%)	1.000
Insomnia	10 (23.8%)	15 (24.6%)	0.928	7 (20.6%)	9 (26.5%)	0.567
Tobacco smoking	12 (28.6%)	8 (13.1%)	0.051	7 (20.6%)	7 (20.6%)	1.000
Alcohol consumption	7 (16.7%)	1 (1.6%)	0.007	1 (2.9%)	1 (2.9%)	1.000
DAO (U/L)	5.10 (2.71–8.24)	4.56 (2.47–9.16)	0.740	5.38 (2.66–10.58)	5.51 (3.26–10.15)	0.556
D-lactate (U/L)	12.60 (6.96–19.90)	12.39 (7.07–17.53)	0.715	11.48 (6.83–19.32)	13.85 (11.12–19.80)	0.257
LPS (U/L)	9.46 (2.58–16.95)	7.08 (3.12–10.53)	0.244	6.26 (2.07–17.38)	9.60 (6.12–12.06)	0.536

Data are expressed as the number (%), mean ± standard deviation, or median (interquartile range). BMI: body mass index; DAO: diamine oxidase; LPS: lipopolysaccharide; WMT: washed microbiota transplantation

**Table 2:** Clinical outcomes

	Probiotics	WMT	*P*
Cure^1^	5 (14.7%)	9 (26.5%)	
Remission^2^	9 (26.5%)	19 (55.9%)	0.002
Ineffectiveness	20 (58.8%)	6 (17.6%)	

^1^Decrease in IBS­-SSS by > 100 points and Grade 4 in the Bristol Stool Form Scale for IBS; ^1^Decrease in the Wexner Scoring Scale score by > 70% and Grade 4 in the Bristol Stool Form Scale for FC; ^1^Grade 4 in the Bristol Stool Form Scale and 1–2 stools per day for FDr; ^1^Decrease in the GSRS score by > 80% for FAB

^2^Decrease in IBS­-SSS by > 75 points or improvement of ≥ 1 grade in the Bristol Stool Form Scale for IBS; ^2^Decrease in the Wexner Scoring System score by 30–69% or an increase ≥ 1 grade in the Bristol Stool Form Scale score for FC; ^2^Decrease in the Bristol Stool Form Scale score by > 1 grade or a reduced frequency of stools per day for FDr; ^2^Decrease in the GSRS score by 50–80% for FAB

**Table 3:** Intestinal mucosal barrier function (U/L)

Group	N	DAO		D-lactate		LPS	
		before	after	Before	after	before	after
Probiotics	34	5.38 (2.66–10.58)	9.02 (4.03–12.30)	11.48 (6.83–19.32)	13.81 (9.40–16.93)	6.26 (2.07–17.38)	9.93 (8.35–11.47)
WMT	34	5.50 (3.26–10.14)	4.66 (3.10–9.80)	13.85 (11.12–19.80)	11.55 (7.51–14.24)	9.60 (6.12–12.06)	10.10 (15.19–11.93)

Data are expressed as the number or median (interquartile range). DAO: diamine oxidase; LPS: lipopolysaccharide

**Table 4:** Multivariate Cox regression analysis for FBDs

	HR	95%Cl	*P*
Age	1.025	0.983–1.068	0.248
Sex	1.212	0.540–2.717	0.641
BMI	1.002	0.864–1.164	0.974
DAO	0.859	0.335–2.200	0.751
D-lactate	0.248	0.093–0.666	0.006
LPS	0.800	0.237–2.700	0.719
Anxiety	1.100	0.143–8.482	0.927
Depression	0.336	0.356–20.94	0.336
Insomnia	0.986	0.365–2.663	0.978
Alcohol consumption	0.584	0.077–4.398	0.601
Tobacco smoking	0.299	0.070–1.273	0.102

## Discussion

Recent evidences showing the important role of intestinal flora in the pathogenesis of FBDs has led to a heightened interest in the use of intestinal microbiota for FBD treatment. The therapeutic potential of FMT for restoring the gut microbiota in IBS and FC has been investigated in randomized clinical trials. However, the results have been inconsistent. FMT has been shown to be superior to placebo in the treatment of IBS [22–24] and FC [25–27]. Moreover, a double-blind, randomized, placebo-controlled, parallel-group, single-center study conducted by Johnsen and colleagues has shown that FMT provided significant symptom relief in patients with IBS [22]. El-Salhy and coworkers recruited 165 patients with IBS to accept FMT randomly (own stools or from donors) and found that patients who received FMT exhibited a significant improvement in fatigue and quality of life [23]. However, some studies have shown that FMT is not superior to placebo [28, 29]. Halkjær and collaborators conducted a clinical trial and revealed that IBS patients in the placebo group experienced greater symptom relief compared with that in the FMT group [28]. Similarly, Aroniadis and colleagues have reported that FMT did not induce significant symptom relief in IBS with diarrhea in comparison to placebo [29]. However, in our study, WMT was found to be more effective than probiotics in the treatment for FBD patients. In our study, WMT resulted in a cure in 26.5% (9/34) and remission in 55.9% (19/34) of the patients, which was markedly better than the corresponding values in the probiotics group (14.7% and 26.5%, respectively).

The discrepancy between the results of ours and previous studies can be explained by three reasons. First, instead of using the weight of a stool, we prepared the washed microbiota with microfiltration using an automatic purification system, followed by repeated centrifugation plus three-time suspension, which enables delivery of a precise dose of the enriched microbiota [19]. Second, preparation of the washed microbiota was completed within 1 h, and therefore, transplantation could be done promptly, with minimal external exposure time. In contrast, in most countries, fecal capsules or frozen stool samples are generally used. Third, fecal microbiota in our study was transplanted to the colon or more proximal portions of the intestine through transendoscopic enteral tubing [30]. This strategy can increase the intestinal contact time of the fecal microbiota and facilitate colonization of the microbiota in the intestinal tract.

Intestinal dysbacteriosis is widely believed to contribute to the reduced function of the intestinal mucosal barrier. Patients with IBS or FC have low levels of beneficial flora (e.g., *Lactobacillus* and *Bifidobacterium* species) and increased levels of potentially pathogenic bacteria [31, 32]. Harmful bacteria remain in the intestinal lumen for a long time, eventually causing direct or indirect damage to the intestinal mucosal barrier. The presence of excessive numbers of pathogenic bacteria leads to the increased production of bacterial toxins that may invade the intestinal mucosa, causing increased mucosal permeability.

Several studies have shown that intestinal permeability can be improved by altering the gut microbiota using therapeutic options such as synbiotics, prebiotics, probiotics, and FMT. For example, a randomized, single-blind, placebo-controlled, pilot trial conducted by Cosola and colleagues in chronic kidney diseases (CKD) patients and healthy controls revealed that treatment with synbiotics for two months resulted in a reduction of small-intestinal permeability and constipation syndromes in the CKD group [33]. Similarly, Ho and colleagues have shown that intestinal permeability can be improved in children with type-1 diabetes mellitus by administration of oligofructose-enriched inulin for 12 weeks [34]. Furthermore, allogenic FMT has been shown to reduce small-intestinal permeability in patients with non-alcoholic fatty liver disease [35]. Wang and collaborators have shown that FMT can alter intestinal permeability in rats with carbon tetrachloride–induced acute hepatic dysfunction [36].

However, a few intervention studies have shown that in FBDs, especially IBS, the intestinal permeability can be modified by altering the gut microbiota. Bonfrate and colleagues have shown that oral administration of bacteria of the genera *Bifidobacterium* and *Lactobacillus* for 2 months restored intestinal permeability and gut microbiota in IBS patients [37]. In addition, use of probiotic-fermented milk for 4 weeks was shown to improve mucosal-barrier function in IBS-D patients in a randomized, single-blind, placebo-controlled study [20].

In our study, serum levels of DAO, D-lactate, and LPS were used to evaluate IBF in patients [38, 39]. Levels of these markers have been shown to increase with an increase in intestinal permeability. Measurements of DAO, D-lactate, and LPS levels before and after treatment revealed a difference in the degree of damage to the intestinal barrier in the probiotics group and WMT group (58.8% vs. 67.6%). IBF was found to have improved in the WMT group with treatment, while no such change was noted in the probiotics group. Ours is the first study to demonstrate that manipulation of the microbiome is associated with an improvement in intestinal permeability in patients with FBDs. Since increased permeability of the intestinal barrier may be involved in the occurrence and development of FBDs, WMT may represent a new method to treat FBDs.

D-lactate is a product of the metabolism and lysis of bacteria. Damage to the IBF causes an increase in intestinal permeability and the D-lactate produced by the intestinal bacteria cross the intestinal mucosa enter circulation. Studies have shown that serum levels of D-lactate are high in patients with severe IBS and that these levels are negatively correlated with levels of *Lactobacillus* and *Bifidobacterium* species [32]. Consistent with these findings, mice with induced IBS have been found to exhibit reduced plasma levels of D-lactate on treatment with *Bifidobacterium* species or *Lactobacillus* species [40]. Consistent with these findings, our study showed that D-lactate levels in FBD patients treated with WMT were significantly lower than those in FBD patients treated with probiotics. Furthermore, multivariate regression analysis of factors that may affect the prognosis of patients with FBDs revealed that increased D-lactate levels were associated with a better outcome. In the future, screening for increased D-lactate levels could be used to select patients likely to benefit from WMT.

Our study had four main limitations. First, the most important bias of PSMA is selection bias. Recall bias and confounding bias are also difficult to avoid in a retrospective study. The bias can affect the validity and authenticity of the results. Second, this was a single-center study with a small sample size. Hence, the statistical power in this study may not be sufficient to detect the effects of WMT on FBDs. Third, since this was a retrospective study, fecal samples were not collected from FBD patients. Hence, the therapeutic effect of WMT on intestinal permeability and intestinal microbiota is not known. Fourth, only plasma levels of DAO, D-lactate, and LPS were used to evaluate the severity of IBF. More accurate parameters are necessary to evaluate IBF.

## Conclusion

To summarize, the results of our retrospective analysis indicate that WMT may improve symptoms and IBF in patients with FBDs. WMT induced a significant reduction in D-lactate levels, and reduced D-lactate levels were found to be associated with a better prognosis for patients with FBDs receiving WMT. Our findings suggest that WMT shows promise as a therapeutic option for the regulation of gut microbiota in FBD.

## Data Availability

The datasets used and analyzed in the current study are available from the corresponding author on reasonable request.
